# Impact of National health insurance on medication adherence among hypertensive patients: A hospital-based cross-sectional study from Kailali, Nepal

**DOI:** 10.1371/journal.pone.0332602

**Published:** 2025-09-19

**Authors:** Nripa Raj Dangaura, Pratik Khanal, Bihari Sharan Kuikel, Sabina Marasini, Ashish Timilsina, Prakash Chandra Joshi, Roshan Mahato, Archana Shrestha, Biraj Man Karmacharya

**Affiliations:** 1 Department of Public Health and Community Programs, Kathmandu University School of Medical Sciences, Dhulikhel, Nepal; 2 Bergen Centre for Ethics and Priority Setting in Health (BCEPS), Department of Global Public Health and Primary Care, University of Bergen, Bergen, Norway; 3 Family Welfare Division, Department of Health Services, Ministry of Health and Population, Nepal; 4 James P Grant School of Public Health, BRAC University, Nepal; 5 Department of Chronic Disease Epidemiology, Yale School of Public Health, New Haven, United States of America; 6 Institute for Implementation Science and Health, Kathmandu, Nepal; Tribhuvan University, NEPAL

## Abstract

**Introduction:**

Noncompliance of medication among patients with chronic disease is a major challenge for the health system. Expanding of national health insurance among patients might increase their access to health care services, reduce out of pocket expenditures, and improve health outcomes. This study aimed to determine the association between enrollment in health insurance and medication adherence among hypertensive patients in Nepal.

**Methods:**

A cross-sectional study was conducted among 402 patients visiting the outpatient department of Tikapur Hospital located in Kailali, Nepal. Data was collected by face-to-face interviews using a structured questionnaire. Adherence to hypertensive medication was assessed using the Hill Bone Medication Adherence Scale (HB-MAS). A multivariable logistic regression model was constructed to ascertain the association between enrollment in health insurance and medication adherence.

**Results:**

Overall, 52.7% (60.2% of uninsured and 45.3% of insured) of the patients was medication adherence. Enrollment in health insurance was not significantly associated with medication adherence. The participants among those who had reported more than secondary level education was higher odds of medication adherence (AOR = 3.30; 95% CI: 1.25–8.73); those who reported more than five minutes of interaction with doctors (AOR = 2.97; 95% CI: 1.56–5.65); those on medication for more than 10 years had higher odds of adherence (AOR:2.56; 95% CI:1.35-4.86); aged group 50-59 years was lower odds of medicatio n (AOR = 0.46; 95% CI: 0.23-0.91); compared to patients with no formal education;those who reported less than or equal to 5 minutes of interaction with doctors; those who had less than 5 years of medication; participants younger than 50 years respectively.

**Conclusion:**

Our study showed that enrollment in health insurance was not associated with medication adherence among patients with hypertension. Health system interventions such as improving counseling, patient education, and follow-up, and ensuring availability of medicines might improve medication adherence among patients. Health professionals also should set up education, and interventions aimed at increased awareness of the consequences of non-adherence to antihypertensive medication.

## Background

Control of blood pressure helps to prevent the occurrence of any adverse health outcomes like coronary heart disease, heart failure, stroke, and premature death [1]. Patients who do not take their antihypertensive medicines as prescribed have a higher risk of adverse consequences such as hospitalization and higher healthcare expenditures due to complications in comparison to those who have good adherence. It is estimated that approximately 50% of hypertensive patients do not take their medications as prescribed [2]. Although people’s knowledge of hypertension has risen as the worldwide burden of the disease has increased, but it is still not adequately treated [3]. One of the key challenges of the health system in developing countries is to maintain good adherence to antihypertensive medications [4]. Chronic diseases need long-term treatment, so adherence to medication is essential for disease management and averting consequences [5].

A 2017 systematic review and meta-analysis reported that 80% of deaths in low-and middle-income countries (LMICs) were due to complications of hypertension, with non-adherence as high as 63% among adults [6]. Clinical trials have proved that individuals getting therapy for chronic illnesses had adherence on an average rate ranging from 43 to 78 percent [7,8]. A cross-sectional study conducted from 12 African countries revealed that 35.6% have poor adherence in African patients [9]. Studies from South Asian countries revealed similar findings. The anti-hypertensive medication adherence was 52%−62% in Nepal [10–12] 50.3% in India [13], 77% in Pakistan [14], and 73.8% in Bangladesh [15]. Medication cost is one of the major factors which results in poor medication adherence among hypertensive patients [12,16]. The World Health Organization (WHO) included antihypertensive medication in the list of essential medicines such as amlodipine, bisoprolol, enalapril, hydralazine, hydrochlorothiazide, and methyldopa so as to improve access to medications among those affected in the member countries [17].

Health insurance is a fundamental tool for achieving equitable access to healthcare care services and promoting public health. Nepal started national health insurance program (NHIP) from Kailali- a district in Sudurpashchim province since 2016. Enrollment in NHIP is family-based and provides financial protection through a comprehensive benefit package with a ceiling of 100,000 Nepalese rupees (equivalent 748.83 US Dollars; 1 USD= ~ 133.54 NPR). The benefit package also provides access to hypertension care including antihypertensive medicines [18]. However, enrollment coverage in NHIP is very low, with only 11% of the total population enrolled in 2019 and the dropout rate of 38% among the enrolled members in 2018 [19]. In 2019, hypertension was the third most common morbidity covered by health insurance in Nepal [20]. Evidence has shown that health insurance helps to control hypertension by increasing access to treatment [21–23]. Likewise, there can be several factors that contribute to poor medication adherence such as socio-demographic factors (sex, marital status), duration of medication, comorbidity, cost of medicine, and irregular follow-up [11,16]. Previous studies have shown mixed results regarding the relationship between health insurance and medication adherence. With the initiation of NHIP in Nepal, not much is known about the role of health insurance in influencing health outcomes such as medication adherence among hypertensive population in Nepal. In this context, this study aims to address this research gap by assessing association between enrollment in health insurance and medication adherence among hypertensive patients.

## Methods

### Study design and study site

A hospital-based cross-sectional study was conducted between April-August 2021 among patients with hypertension visiting Tikapur Hospital. This hospital is in Kailali district of Sudurpashchim province of Nepal and is one of the hospitals where health insurance was first implemented. This hospital provides basic health services, emergency services, dental services, and OPD services and has a good flow of hypertensive patients. The hospital has been implementing the NHIP since 2016 [24].

### Participants

We collected data from convenience samples of 402 hypertensive patients who were at least 18 years or older; either insured at least 1-year or uninsured in the NHIP; currently taking medication for at least six months and ability to take medication without assistance. The sample size was estimated with 80% power and 95% confidence interval to detect 56% medication adherence among uninsured and 70% medication adherence among insured hypertensive patients, adjusting for 10 percent non-response [12,25,26].

### Data collection

Trained research assistants interviewed 201 insured and 201 uninsured participants in Nepali language. These patients visited outpatient department of the hospital for their follow-up treatment. Ethical approval of the study was obtained from the Institutional Review Committee of Kathmandu University School of Health Sciences (Reference Number: 125/20-IRC, approval date: 25th November 2020).

### Measures

#### Medication adherence.

We measured medication adherence using the nine item Hill bone medication adherence scale [27]. The tool was translated into Nepali language and was back translated by independent researchers. We pretested the tool among the 10 participants from each insured and uninsured group and calculated Cronbach’s alpha (0.67) to determine the reliability [28].

Medication Adherence Scale had a four-point Likert-type response format for each question. Each response carried a score: all of the time = 1, most of the time = 2, some of the time = 3 and none of the time = 4. The total scores were added for each patient with a range from 9 (minimum) to 36 (maximum). Lower scores reflected poorer adherence whereas a higher score meant better medication therapy. However, as the scale does not prescribe any cut-off points, we calculated the median score for the cut-off point to perform multivariable logistic regression [29]. The distribution of scores was skewed to the left (Skewness −0.99, kurtosis 4.58) with a median score of 35 (IQR 32–36), indicating more patients scoring high points were defined as adherence. A score of 34 and below was categorized as non-adherence.

#### Socio-demographic and clinical variables.

The information on socio-demographic variables included participant’s age (completed years); sex (Male/Female); ever gone to school (Yes/No); marital status (Never married/ Currently married/ Separated/ Divorced/ Widowed/ Cohabiting); and highest level of education status (No formal education/ Under primary school/ Primary School completed/ Secondary School completed/ Intermediate school (10 + 2)/ Bachelors completed/ Masters or above); religion (Hindu/ Buddhist/ Muslim/ Christian/Kirat/ Others); residence (urban/rural); income monthly (Nrs.) divided in quartiles, from first quartile (Q1-being poorest) to forth quartile (Q4-being wealthiest) each quartile containing approximately 25% of the population; ethnicity (Dalit/ Janajati (excluding Tharu)/ Madhesii/ Muslim/ Brahmin/ Chhetri/ Tharu/ Others); time to reach health facility (Minutes); one-way transportation fee (Nrs.).

The information on clinical and other variables were also collected such as duration of hypertension (years); duration of medication intake (months); number of medicines; family history of hypertension (Yes/No); comorbidity (Yes/No); number of health facility visit/month; average time of OPD check-up (minutes); attendance on counseling of hypertension (Yes/No) and average monthly expense of hypertension medicines (Nrs.) [30–32].

### Data analysis

Numerical data were reported in means and standard deviation, and categorical data with frequency and percentage. We applied binary and multivariable logistic regression models to determine the association between enrollment in health insurance and medication adherence among hypertensive patients. We represented bivariate and multivariate logistic regression results using crude odds ratios (COR), adjusted odds ratios (AOR), their 95% confidence intervals and considered p-values less than 0.05 to be statistically significant. The variables that were fitted into the multivariable regression model were enrollment in health insurance (main predictor variable) and other covariates that included sex, age, religion, education, ethnicity, marital status, occupation, time to reach health facility, duration of medication, family history of hypertension, comorbidity, number of visits to the health facility, duration of interaction with doctor, and counseling of hypertension. All the socio-demographic and clinical variables were included as covariates to control for their potential confounding effects. To ensure the reliability of the model estimates, we checked for multicollinearity using variance inflation factor and evaluated the goodness-of-fit of the model using Hosmer-Lemeshow test. All analyses were conducted using STATA version 13.0 (Stata Corp, College Station, Texas, USA) for cleaning, coding and statistical analysis.

## Results

### Socio-demographic characteristics

Table 1 shows the socio-demographic characteristics of participants. Among 402 hypertensive patients, 48% were males. The mean age of the study participants was 61.2 ± 12.4 years. Of the total participants, majority were Hindu (98%). The educational attainment also varied among participants where the majority (68%) had no formal education. Majority (75%) were married, about one third (30%) were of Tharu ethnicity, and 62% of the participants were unemployed. About 71% of participants reported that they reached the health facility within 30 minutes.

**Table 1:** Socio-demographic characteristic of the insured and uninsured participants.

Characteristics	Total participant (n = 402)	Uninsured (n = 201)	Insured (n = 201)
	N (%)	N (%)	N (%)
**Sex**			
Female	209 (52.0)	97 (48.3)	112 (55.7)
Male	193 (48.0)	104 (51.7)	89 (44.3)
**Age (Mean±SD)**	61.2 ± 12.4	57.8 ± 12.1	64.5 ± 11.9
<50	73 (18.2)	55 (27.4)	30 (14.9)
50-59	101 (25.1)	69 (34.3)	40 (19.9)
60-69	95 (23.6)	32 (15.9)	43 (21.4)
70+	133 (33.1)	45 (22.4)	88 (43.8)
**Religion**			
Non-Hindu	11 (2.7)	7 (3.5)	4 (2.0)
Hindu	291 (97.3)	194 (96.5)	197 (98.0)
**Education**			
No formal education	273 (67.9)	120 (59.7)	153 (76.1)
Primary	62 (15.4)	36 (17.9)	26 (12.9)
Secondary	27 (6.7)	16 (8.0)	11 (5.5)
More than Secondary	40 (10.0)	29 (14.4)	11 (5.5)
**Ethnicity**			
Tharu	121 (30.1)	61 (30.3)	60 (29.8)
Chhetri	115 (28.6)	57 (28.4)	58 (28.9)
Brahmin	65 (16.2)	34 (16.9)	31 (15.4)
Janajati & Others	20 (5.0)	8 (4.0)	12 (6.0)
Dalit	81 (20.1)	41 (20.4)	40 (19.9)
**Marital Status**			
Married	301 (74.9)	152 (75.6)	149 (74.1)
Others^1^	101 (25.1)	49 (24.4)	52 (25.9)
**Occupation**			
Unemployed^2^	250 (62.2)	105 (52.2)	145 (72.1)
Employed	152 (37.8)	96 (47.8)	56 (27.9)
**Income Monthly Quartiles (Nrs.)**			
Q1 (Lowest Quartile)	113 (33.1)	55 (31.6)	58 (34.7)
Q2	83 (24.3)	40 (23.0)	43 (25.7)
Q3	106 (31.1)	61 (35.1)	45 (27.0)
Q4	39 (11.5)	18 (10.3)	21 (12.6)
**Time to reach Health Facility (Min.)**			
<30	286 (71.1)	130 (64.7)	156 (77.6)
≥30	116 (28.9)	71 (35.3)	45 (22.4)
**Medication Adherence**			
No	190 (47.3)	80 (39.8)	110 (54.7)
Yes	212 (52.7)	121 (60.2)	91 (45.3)

^*1*^*Never married/Separated/widowed* ^*2*^*Students/Retired/Housewife/others*

### Medication adherence of the insured and uninsured participants by socio-demographic characteristics

Table 2 presents the socio-demographic characteristics of the insured and uninsured participants based on their medication adherence status. Among the females, 57.7% of uninsured and 41.1% of the insured females were adherent to antihypertensive medication. Similarly, 62.5% of uninsured males and 50% of the insured males were adherent. Almost all age groups had more than 50% adherence among the uninsured participants. In contrast, only less than 50 years and more than 70 years participants had at least 50% adherence in insured groups. The proportion of medication adherence was highest in the participants who had more than secondary education in both uninsured (75.9%) and insured (81.8%) participants. Among uninsured , all ethnic groups, except Dalit, had more than 50% adherence, whereas none of the ethnic groups in the insured group had more than 50% medication adherence. Married participants, compared to unmarried participants, had a higher proportion of medication adherence in both uninsured (62.5% vs. 53.1%) and insured groups (46.3% vs. 42.3%). Medication adherence was similar across both unemployed (60.0%) and employed groups (60.4%) in the uninsured participants whereas it was higher in employed (60.7%) participants compared to unemployed (39.3%) in the insured participants. Among the uninsured participants, the proportion of medication adherence was almost similar across those who reported duration to health facility as less than 30 minutes (59.2%) and 30 minutes or more (62%), whereas the proportion of adherence was higher in those who reported 30 minutes or more in insured participants (62.2% vs. 40.4%).

**Table 2:** Medication adherence of the insured and uninsured participants by socio-demographic characteristics.

	Uninsured (n = 201)			Insured (n = 201)		
	N (%)	Non-Adherent n (%)	Adherent n (%)	Participant N (%)	Non-Adherent n (%)	Adherent n (%)
**Sex**						
Female	97 (48.3)	41 (42.3)	56 (57.7)	112 (55.7)	66 (58.9)	46 (41.1)
Male	104 (51.7)	39 (37.5)	65 (62.5)	89 (44.3)	44 (49.4)	45 (50.6)
**Age Category**						
<50	55 (27.4)	20 (36.4)	35 (63.6)	30 (14.9)	12 (40.0)	18 (60.0)
50-59	69 (34.3)	35 (50.7)	34 (49.3)	40 (19.9)	26 (65.0)	14 (35.0)
60-69	32 (15.9)	10 (31.3)	22 (68.7)	43 (21.4)	28 (65.1)	15 (34.9)
70+	45 (22.4)	15 (33.3)	30 (66.7)	88 (43.8)	44 (50.0)	44 (50.0)
**Religion**						
Non-Hindu	7 (3.5)	4 (57.1)	3 (42.9)	4 (2.0)	3 (75.0)	1 (25.0)
Hindu	194 (96.5)	76 (39.2)	118 (60.8)	197 (98.0)	107 (54.3)	90 (45.7)
**Education**						
No formal education	120 (59.7)	47 (39.2)	73 (60.8)	153 (76.1)	85 (55.6)	68 (44.4)
Primary	36 (17.9)	20 (55.6)	16 (44.4)	26 (12.9)	17 (65.4)	9 (34.6)
Secondary	16 (8.1)	6 (37.5)	10 (62.5)	11 (5.5)	6 (54.5)	5 (45.5)
More than Secondary	29 (14.3)	7 (24.1)	22 (75.9)	11 (5.5)	2 (18.2)	9 (81.8)
**Ethnicity**						
Tharu	61 (30.4)	24 (39.3)	37 (60.7)	60 (29.8)	31 (51.7)	29 (48.3)
Chhetri	57 (28.4)	16 (28.1)	41 (71.9)	58 (28.9)	30 (51.7)	28 (48.3)
Brahmin	34 (16.9)	16 (47.1)	18 (52.9)	31 (15.4)	17 (54.8)	14 (45.2)
Janajati & Others	8 (3.9)	3 (37.5)	5 (62.5)	12 (6.0)	9 (75.0)	3 (25.0)
Dalit	41 (20.4)	21 (51.2)	20 (48.8)	40 (19.9)	23 (57.5)	17 (42.5)
**Marital Status**						
Married	152 (75.6)	57 (37.5)	95 (62.5)	149 (74.1)	80 (53.7)	69 (46.3)
Others^1^	49 (24.4)	23 (46.9)	26 (53.1)	52 (25.9)	30 (57.7)	22 (42.3)
**Occupation**						
Unemployed^2^	105 (52.2)	42 (40.0)	63 (60.0)	145 (72.1)	88 (60.7)	57 (39.3)
Employed	96 (47.8)	38 (39.6)	58 (60.4)	56 (27.9)	22 (39.3)	34 (60.7)
**Time to reach HF (min.)**						
<30	130 (64.7)	53 (40.8)	77 (59.2)	156 (77.6)	93 (59.6)	63 (40.4)
≥30	71 (35.3)	27 (38.0)	44 (62.0)	45 (22.4)	17 (37.8)	28 (62.2)

^*1*^*Never married/Separated/widowed* ^*2*^*Students/Retired/Housewife/others*

### Association between medication adherence and health insurance

Enrollment in health insurance was not significantly associated with medication adherence (AOR: 0.66; 95% CI: 0.39–1.10) in hypertensive participants after adjusting for sex, age, religion, ethnicity, education, marital status, occupation, time to reach health facility, duration of medication, family history, comorbidity, number of visit to the health facility, duration of doctor- patient interaction, and counseling of hypertension received. Covariates such as age group, education, duration of medication, and doctor-patient interaction were significantly associated with medication adherence in the study participants.

The study participants between 50–59 years age group were 54% less likely to be adherent to antihypertensive medication compared to participants younger than 50 years (AOR: 0.46; 95% CI:0.23–0.91). The participants who had more than secondary level education had three times higher odds of adherence compared to those who had no formal education (AOR:3.30; 95% CI:1.25–8.73). Patients who had anti-hypertensive medication for more than 10 years had higher odds of adherence compared to those having less than 5 years of medication (AOR:2.56; 95% CI:1.35–4.86). In addition, we found that patients reporting more than 5 minutes of interaction with doctors had almost three times higher odds of adherence compared to those patients who reported less than or equal to 5 minutes (AOR:2.97; 95% CI:1.56–5.65) (Table 3).

**Table 3:** Multivariable logistic regression analysis to determine the association between enrollment in health insurance and medication adherence among hypertensive participants.

Characteristics	Bivariate analysis			Multivariable regression analysis		
	COR	95% CI	p-value	AOR	95% CI	p-value
**Sex (Ref: Female)**						
Male	1.4	0.94-2.06	0.10	1.15	0.68-1.95	0.59
**Age Category (Ref: < 50 years)**						
50-59	0.48	0.27-0.85	0.01	0.46	0.23-0.91	0.02
60-69	0.59	0.31-1.10	0.10	0.60	0.28-1.30	0.20
70+	0.76	0.43-1.32	0.33	1.71	0.54-2.56	0.67
**Religion (Ref: Non-Hindu)**						
Hindu	1.98	0.57-6.90	0.28	1.43	0.36-5.79	0.69
**Education (Ref: no formal education)**						
Primary	0.63	0.36-1.11	0.11	0.60	0.31-1.17	0.13
Secondary	1.17	0.53-2.59	0.69	1.31	0.49-3.47	0.60
More than Secondary	3.22	1.48-7.03	0.003	3.30	1.25-8.73	0.02
**Ethnicity (Ref: Tharu)**						
Chhetri	1.25	0.75-2.09	0.39	1.10	0.60-2.00	0.76
Brahmin	0.81	0.44-1.48	0.49	0.71	0.35-1.45	0.35
Dalit & Others	0.67	0.39-1.13	0.14	0.79	0.43-1.46	0.46
**Marital Status (Ref: Married)**						
Others^1^	0.76	0.48-1.19	0.23	0.71	0.40-1.24	0.23
**Occupation (Ref: Unemployed**^**2**^)						
Employed	1.66	1.10-2.50	0.02	1.05	0.58-1.88	0.88
**Time to reach Health Facility (Ref: < 30 mins))**						
≥30 mins	1.71	1.09-2.65	0.02	1.09	0.64-1.88	0.74
**Duration of Medication Ref (<5 years)**						
5–9 years	1.2	0.78-1.85	0.4	1.28	0.78-2.09	0.33
10 + years	1.88	1.06-3.32	0.03	2.56	1.35-4.86	0.004
**Family History (Ref: No)**						
Yes	0.68	0.46-1.02	0.06	0.65	0.41-1.00	0.06
**Comorbidity (Ref: No)**						
Yes	0.96	0.63-1.46	0.83	0.91	0.55-1.49	0.70
**No. of health facility visit/month (Ref: 1 visit)**						
1 + visit	2.15	1.32-3.52	0.002	1.25	0.68-2.28	0.47
**Duration of doctor-patient interaction (Ref: **≤ **5 mins)**						
>5 mins	3.17	1.96-5.11	<0.0001	2.97	1.56-5.65	0.001
**Counseling of hypertension (Ref: No)**						
Yes	2.57	1.16-5.67	0.02	2.10	0.83-5.31	0.12
**Health Insurance (Ref: No)**						
Yes	0.55	0.37-0.81	0.003	0.66	0.39-1.10	0.11

^*1*^*Never married/Separated/widowed* ^*2*^*Students/Retired/Housewife/others*

## Discussion

We examined the association between enrollment in health insurance and medication adherence among hypertensive patients. Our study found that health insurance was not significantly associated with medication adherence among hypertensive participants. Instead, significant covariates included age, education, duration of medications and duration of doctor-patients relationship.

Similar to our findings, a study conducted in United States showed that there was no difference in odds of blood control on the hypertensive insured patients [33]. Likewise, repeated cross-sectional survey conducted in Mexico also revealed that medication adherence was not significantly associated with health insurance [34]. A study from Ghana found no variation in the utilization of maternity care between women registered in the health insurance [35]. Another study from Ghana showed that medication non-adherence was partly due to prescribed drugs not being covered by the national health insurance, leading to out-of-pocket payments [36]. Therefore, our findings corroborate with findings from other studies showing that there was no significant association between medication adherence and health insurance. The possible reason for this might be that health insurance enrollment alone does not significantly impact medication adherence. In Nepal, the NHIP is characterized by low enrollment, high dropout rates, and poor insuree satisfaction with services at health facilities [37–40]. An exploratory study from Nepal found that high utilization of medical services, especially among patients with chronic diseases, along with delays in purchasing medicines and supplies, led to insufficient availability of medicines [19]. The participants in our study also stated about the frequent unavailability of antihypertensive drugs in the health facility. Thus, the lack of availability of medicines may have a negative impact on medication adherence.

Still, different studies have shown that medication adherence is significantly associated with health insurance. A study from USA showed that patients with health insurance have increased odds of achieving optimal blood pressure control compared to patients without health insurance [23]. Another cross-sectional study conducted in China revealed that medication adherence was associated with health insurance [41]. In a population-based study conducted in Mexico, insured hypertensive adults had a significantly higher probability of receiving antihypertensive treatment [42]. The difference in study findings might be due to the different study settings, design, participants, and sample size. The countries also tend to have strong and different health insurance systems with multiple health insurance providers.

Our study found that the level of adherence to antihypertensive medication was 52%. Similar findings were observed in previous studies conducted in Dhulikhel hospital and Dharan municipality of Nepal where the level of adherence was 51.9% and 56.5%, respectively [11,12]. A study conducted in India reported adherence of 51.3% among 608 hypertensive patients [13]. A systematic review that included low-income countries found that the average proportion of medication adherence was 52.7% [43]. However, higher level of medication adherence were reported in other studies conducted in Nepal (62.1%), Pakistan(77%), Ghana and Nigeria (66.7%) [10,14,44]. Possible explanations for the variation in adherence rates include higher healthcare costs, pharmaceutical costs, patient education on medication adherence, and improved care services.

In our study, patients of aged group 50–59 years had significantly lower odds of medication adherence than those below 50 years. Studies conducted elsewhere show that medication adherence increase with age [14,45,46] which was in contrast to our study findings.

This study revealed that there was a significant association between medication adherence and higher education of study participants. Similar finding was reported from a study done in Jordan [47]. Patients having higher education are likely to make accurate decisions and appropriate lifestyle modifications which may motivate them to maintain compliance with medications.

Communication between healthcare personnel and patients significantly influences the patient’s attitude towards their illness [48]. Our study findings indicate that patients who spent more time in communication with their healthcare providers were more adherent to their medication regimens. Additionally, a meta-analysis conducted by Zolnierek et al. revealed a 19% higher rate of non-adherence among patients reporting poor physician-patient communication [49]. Patients not only expect their primary care physicians to diagnose and treat them effectively but also to excel in contact and communication. They find value in conversing with their physicians, with some viewing it as therapeutic on its own [50].

In this study, respondents with a longer duration of medication were more likely to adhere to anti-hypertensive medication. Similar studies conducted in Malaysia, Pakistan and China also showed that patients diagnosed earlier with hypertension were less likely to maintain compliance when the duration of medication use were shorter. [29,48,49,51,52]. The possible reason may be long-term use of antihypertensive medicines makes patients aware of the consequences of missing doses and develops a positive attitude toward hypertension therapy, both of which motivate patients to remain consistent with their regimen.

### Strengths and limitations

To our knowledge, this is the first study from Nepal to ascertain whether enrollment in national health insurance improves medication adherence among hypertensive patients. Medication adherence was measured with the standardized tool nine-item Hill Bone Medication Adherence Scale. The license was obtained and translated to use in the Nepali version. The sample size was calculated sufficiently with 80% powered to infer the information about social health insurance and medication adherence. Data was collected by trained health professionals with the standardized questionnaire adopted from demographic health survey and WHO Steps Survey.

There are some limitations. First, because it was a cross-sectional study, we were unable to establish temporality and causality in relation to national health insurance and medication adherence. Second, the information on income could not be used due to large amounts of missing data which is a potential confounder. Third, generalization cannot be done due to the sampling method and study of the population as the study was done in one hospital, which did not represent every other characteristic of the Nepalese hypertensive population. Fourth, medication adherence was self-reported, and we did not apply drug visual aids or packing verification, the findings thus could have been affected by social desirability or recall bias. Since, the study was conducted in a hospital-based setting, the participants are self-selected group and hence might not accurately reflect the adherence status in population level. Additionally, the study did not use qualitative information to explore issues regarding non-adherence and did not involve the perspectives of service providers.

## Conclusion

Our study showed enrollment in health insurance was not significantly associated with medication adherence among patients with hypertension. Other factors such as lower age, more than secondary level of education, higher duration of medication and longer duration of doctor-patient interaction were associated with higher medication adherence. This finding provides beneficial information to the health system stakeholders engaged in improving blood pressure control Based on the study findings, we recommend implementing educational interventions to improve health literacy about hypertension, enhancing provider-patient interaction, and providing continuous support and follow-up for patients.

## Supporting information

S1 File Final data set. (XLSX)
